# Frailty assessed by administrative tools and mortality in patients with pneumonia admitted to the hospital and ICU in Wales

**DOI:** 10.1038/s41598-021-92874-w

**Published:** 2021-06-28

**Authors:** Tamas Szakmany, Joe Hollinghurst, Richard Pugh, Ashley Akbari, Rowena Griffiths, Rowena Bailey, Ronan A. Lyons

**Affiliations:** 1 Department of Anaesthesia, Intensive Care and Pain Medicine, Division of Population Medicine, Cardiff University, UHW B Block 3, Heath Park Campus, Cardiff, CF14 4XN UK; 2 Critical Care Directorate, Grange University Hospital, Aneurin Bevan University Health Board, Cwmbran, UK; 3 Population Data Science and Health Data Research UK (HDR-UK), Swansea University, Swansea, UK; 4 Department of Anaesthetics, Glan Clwyd Hospital, Betsi Cadwaladr University Health Board, Rhyl, UK

**Keywords:** Respiratory tract diseases, Risk factors, Epidemiology, Outcomes research

## Abstract

The ideal method of identifying frailty is uncertain, and data on long-term outcomes is relatively limited. We examined frailty indices derived from population-scale linked data on Intensive Care Unit (ICU) and hospitalised non-ICU patients with pneumonia to elucidate the influence of frailty on mortality. Longitudinal cohort study between 2010–2018 using population-scale anonymised data linkage of healthcare records for adults admitted to hospital with pneumonia in Wales. Primary outcome was in-patient mortality. Odds Ratios (ORs [95% confidence interval]) for age, hospital frailty risk score (HFRS), electronic frailty index (eFI), Charlson comorbidity index (CCI), and social deprivation index were estimated using multivariate logistic regression models. The area under the receiver operating characteristic curve (AUC) was estimated to determine the best fitting models. Of the 107,188 patients, mean (SD) age was 72.6 (16.6) years, 50% were men. The models adjusted for the two frailty indices and the comorbidity index had an increased odds of in-patient mortality for individuals with an ICU admission (ORs for ICU admission in the eFI model 2.67 [2.55, 2.79], HFRS model 2.30 [2.20, 2.41], CCI model 2.62 [2.51, 2.75]). Models indicated advancing age, increased frailty and comorbidity were also associated with an increased odds of in-patient mortality (eFI, baseline fit, ORs: mild 1.09 [1.04, 1.13], moderate 1.13 [1.08, 1.18], severe 1.17 [1.10, 1.23]. HFRS, baseline low, ORs: intermediate 2.65 [2.55, 2.75], high 3.31 [3.17, 3.45]). CCI, baseline < 1, ORs: ‘1–10′ 1.15 [1.11, 1.20], > 10 2.50 [2.41, 2.60]). For predicting inpatient deaths, the CCI and HFRS based models were similar, however for longer term outcomes the CCI based model was superior. Frailty and comorbidity are significant risk factors for patients admitted to hospital with pneumonia. Frailty and comorbidity scores based on administrative data have only moderate ability to predict outcome.

## Introduction

Outcomes from critical illness among older patients and those with poorer health status are of increasing significance as global populations age, and have been the subject of intense interest during the SARS-CoV-2 pandemic^1,2^. Frailty is more common with increasing age and is often defined as a syndrome of physiological decline, characterised by marked vulnerability to adverse health outcomes^3,4^. Frailty has been shown to modify the treatment effect of multiple high-risk interventions and independently predicts adverse outcomes beyond traditional comorbidities in several populations, including pneumonia^5^.

The Clinical Frailty Scale^6^ has been widely adopted in the acute setting as an easily applied judgement-based tool, and is specifically referenced within national COVID-19 decision-making guidance^7^. It is often considered to be a composite assessment, which explicitly incorporates disability and the consequences of comorbidity^8^. However, there is inherent subjectivity in such assessment, and we have previously described the limits to inter-rater reliability, and the need for training to enable consistent application^9^. Overall, there remains some uncertainty about how best to identify frailty in relation to acute and critical illness^10,11^.

Alternative approaches to the identification of frailty which adopt a cumulative deficit model have recently been applied to UK National Health Service (NHS) electronic primary care data, the electronic frailty index (eFI)^12^, and to hospital records, the Hospital Frailty Risk Score (HFRS)^13^, but these measures have not been fully evaluated in a critical care setting. Pneumonia has historically been a major cause of mortality^14,15^, a leading cause of sepsis, and one of the most common indications for critical care admission^16^. A very recent US study indicated significant association of HFRS with 30-day mortality in older patients hospitalised with pneumonia^5^, however data is currently limited regarding the predictive validity of frailty indices in relation to longer-term outcomes from critical illness.

In this study we aim to answer the following question: how is patient frailty and comorbidity, as identified by administrative tools, associated with inpatient, 6-month and 1-year mortality following hospitalisation with pneumonia?

## Methods

### Data sources

We used the Secure Anonymised Information Linkage (SAIL) Databank (www.saildatabank.com) to investigate short and long-term mortality in patients admitted with pneumonia to Welsh National Health Service (NHS) hospitals. The development of the SAIL Databank as a secure privacy protecting Trusted Research Environment (TRE) of anonymised person-based records has been described previously^17,18^. The analysis of anonymised linked data was approved by the independent Information Governance Review Panel (IGRP) of the SAIL Collaboration Review System (Longitudinal analysis of Critical Care Outcomes in Wales, Project No: 0634, 20/06/2017).

We utilised the following data sources from the SAIL Databank in this retrospective cohort study: the Welsh Critical Care Dataset (CCDS, collated from the monthly exports of the Critical Care Minimum Dataset from all Welsh ICUs—including organ support, admission and discharge data), the Welsh Demographic Service Dataset (WDSD, demographic data submitted by primary care services), the Patient Episode Database for Wales (PEDW), Welsh Longitudinal General Practice (WLGP), and the Annual District Death Extract (ADDE) of the Office for National Statistics (ONS).

### Study cohort

We used the PEDW inpatient data to identify patients admitted to hospital for the first time with a diagnosis of pneumonia or flu between 1st January 2010 and 31st December 2018. A pneumonia or flu diagnosis was defined using the International Coding of Disease version 10 (ICD-10) codes J09-J18. This data was linked with the WDSD to obtain the week of birth, sex and Lower Super Output Area (LSOA) of patient residence version 2011 (each LSOA contains approximately 1500 people) and onward linked to Welsh Index of Multiple Deprivation (WIMD) 2019 of each patient. We used week of birth to generate age on the admission date and included all patients aged 18-years or more. We identified patients with an ICU admission as those with an in-patient spell from PEDW that also contained a spell recorded in CCDS. Furthermore, we limited the cohort to those with high quality matching from the linkage and anonymisation process as previously described^18^.

### Assessment of comorbidity and frailty

#### Hospital frailty risk score

The Hospital Frailty Risk Score (HFRS) was developed using Hospital Episode Statistics (a database containing details of all admissions, Accident and Emergency (A&E) attendances and outpatient appointments at NHS hospitals in England), and validated on over 1-million older people admitted to hospitals in 2014/15^13^. The HFRS uses ICD-10 codes to search for specific conditions from secondary care. A weight is then applied to the conditions and a cumulative sum is used to determine a frailty status of: Low, Intermediate or High^13^. We calculated the HFRS using PEDW on the date of admission, with a 2-year look back of all hospital admissions occurring in Wales.

#### electronic Frailty Index (eFI)

The eFI is based on an internationally established cumulative deficit model^19^, and assigns a frailty score to an individual calculated using 36 variables from primary care data including symptoms, signs, diseases, disabilities and abnormal laboratory values, referred to as deficits^12^. The eFI score is the number of deficits present, expressed as an equally weighted proportion of the total. An individual with a single deficit would be assigned an eFI of 1/36 (0.03); another with nine deficits would be assigned an eFI of 9/36 (0.25). The eFI score is used to categorise individuals as: fit (eFI value of 0–0.12), mild (> 0.12–0.24), moderate (> 0.24–0.36), or severely frail (> 0.36)^12^. We calculated the eFI retrospectively on the date of admission to hospital using 10-years of previous WLGP data for each individual. As the eFI requires primary care data to be calculated, we restricted the analyses to only patients with a recorded WLGP registration during the hospital spell.

#### Charlson Comorbidity Index (CCI)

The modified CCI was generated using the ICD-10 codes and weights detailed in the NHS Summary Hospital-level Mortality Indicator specification^20^. ICD-10 codes relating to human immunodeficiency virus (HIV) are redacted from the data available within SAIL in accordance with SAIL policies, all other hospital admissions recorded in PEDW in the year preceding the discharge date were included in the CCI calculation^21^. Scores were categorised into three groups: low (-1–0), medium (1–10) and high (> 10).

### Outcomes of interest

The primary outcome was in-patient mortality. The secondary outcomes were 6-month and 1-year mortality. We compared outcomes between those who had been admitted to ICU during the index hospitalisation and those who had not.

### Statistical analysis

Basic demographic data are presented as counts, percentages and figures. To determine if the eFI, HFRS and CCI identify the same groups of individuals, we calculated the categorical scores of both tools for each patient on the day before their index admission date. We then used Chi-squared tests to determine if the tools were associated, and Cramer’s V to determine the strength of association. To be consistent with our methodology at specified time-points and determine associations we calculated multilevel logistic regression models for inpatient, 6-month, and 1-year mortality using a logit link function with mortality as the binary dependent variable. We created separate models for the eFI, HFRS and CCI. In each of the models we included the following variables as fixed effects: ICU admission, eFI (Fit, Mild, Moderate, Severe), HFRS (Low, Intermediate, High), CCI (< 1, 1–10, > 10), age (18–49, 50–59, 60–69, 70–79, 80 +), and sex (Male/Female). We included a random intercept for each admission year to account for potential changes in recording. Statistical analysis was performed using R version 4.0.0 and R2MLwiN^22^. The best fitting logistic regression models were determined using the Area Under the receiver operating characteristic Curve (AUC).

## Results

### Participants

**Figure 1:**
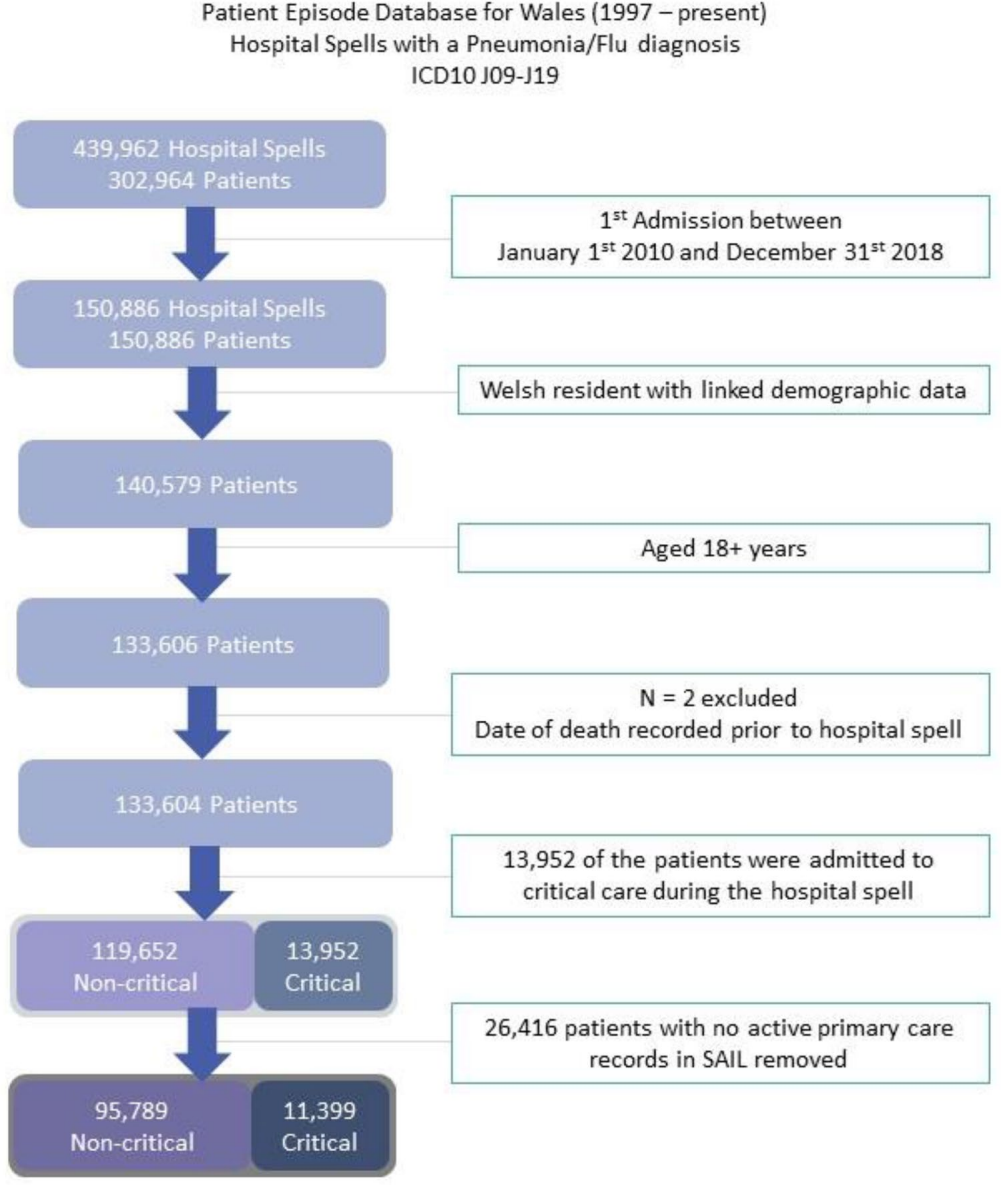
CONSORT diagram for the number of patients in the study. *eFI* electronic Frailty Index; *HFRS* Hospital Frailty Risk Score; *ICU* Intensive Care Unit; *SAIL* (Secure Anonymised Information Linkage) Databank.

There were 133,604 index admissions for patients with pneumonia or flu to Welsh NHS hospitals over the study period. As the eFI is calculated using primary care records the cohort was restricted to only include those registered with a general practice contributing data to SAIL in the study period (80%), this limited the cohort to 107,188 patients. The following flowchart demonstrates how the data for the ICU and hospital cohort was obtained (Fig. 1). Demographic and outcome distributions for the cohorts with and without general practice data are included in Table S1.

### Demographics and distribution of frailty and comorbidity according to eFI, HFRS and CCI

**Figure 2:**
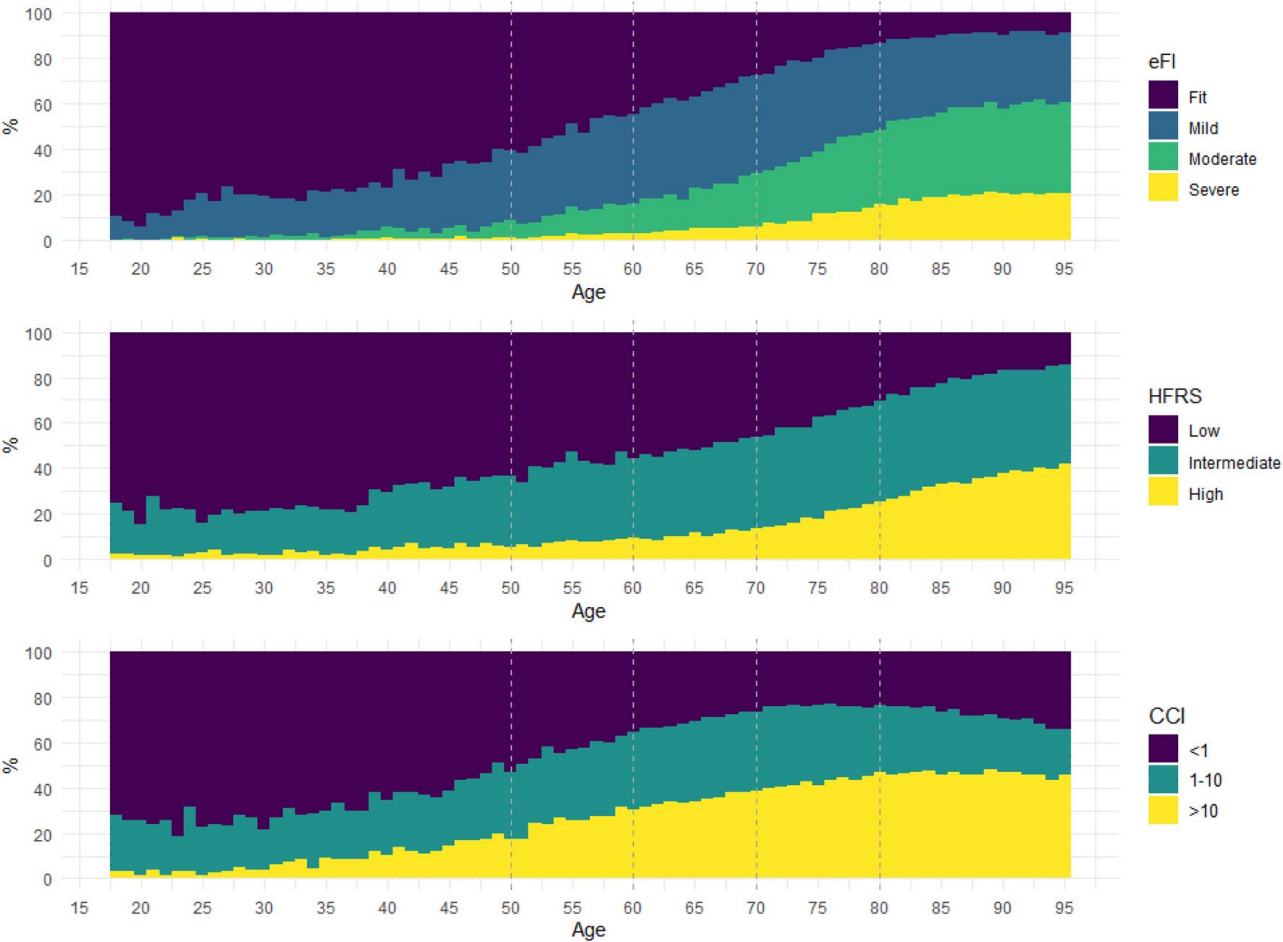
Distribution of frailty and comorbidity categories by age in the cohort. *eFI* electronic Frailty Index; *HFRS* Hospital Frailty Risk Score; *CCI* Charlson comorbidity Index.

The demographic details of the patients are presented in Table 1. The non-ICU hospital admissions were dominated by those aged 80 years and over, whereas 50% of critical care patients were in the 60–79 year group. Higher levels of frailty defined by the eFI were seen in the non-ICU group, and only 23% of the ICU group were considered moderately or severely frail. Whereas the HFRS categorised a larger proportion of individuals as low risk in the non-ICU cohort, most ICU patients were considered “intermediate” risk. There were statistically significant, but clinically irrelevant differences in chronic illness prevalence, as quantified by the CCI, between ICU and non-ICU patients. Interestingly, patients with high comorbidity burden were represented identically between the ICU and non-ICU groups. ICU admissions were more likely to be from deprived areas compared with non-ICU admissions. We also found an increase in frailty (eFI and HFRS) and comorbidities (CCI) with age (Fig. 2).

**Table 1:** Cohort characteristics for the total population and stratified by patients admitted to an Intensive Care Unit (ICU) or not.

		Total	Hospital admission	ICU admission	p-values
	N	107,188	95,789	11,399	
Sex	Female	50.0%	50.8%	44.0%	< 0.001
	Male	50.0%	49.2%	56.0%	
Age (years)	Mean (SD)	72.6 (16.6)	73.5 (16.5)	65.0 (15.7)	< 0.001
Age categories (years)	18–49	10.5%	9.8%	16.5%	< 0.001
	50–59	8.5%	7.7%	14.7%	
	60–69	15.4%	14.4%	23.7%	
	70–79	24.5%	24.2%	27.3%	
	80 +	41.1%	43.9%	17.7%	
Electronic Frailty Index	Fit	26.8%	25.4%	38.7%	< 0.001
	Mild	35.8%	35.5%	38.0%	
	Moderate	26.3%	27.3%	18.0%	
	Severe	11.1%	11.8%	5.2%	
Hospital Frailty Risk Score	Low	38.8%	40.1%	27.3%	< 0.001
	Intermediate	40.1%	38.0%	57.4%	
	High	21.1%	21.8%	15.4%	
Charlson Comorbidity Index	< 1	32.5%	32.6%	31.6%	0.040
	1–10	29.7%	29.6%	30.6%	
	˃10	37.8%	37.8%	37.8%	
WIMD 2019	1. Most deprived	23.6%	23.3%	25.6%	< 0.001
	2	22.2%	22.1%	22.9%	
	3	18.9%	18.9%	18.6%	
	4	17.5%	17.6%	16.5%	
	5. Least deprived	17.8%	18.0%	16.3%	
Mortality	In-patient	23.9%	22.8%	33.5%	< 0.001
	6-months	37.9%	37.6%	40.7%	< 0.001
	1-year	43.7%	43.6%	44.5%	0.066

Chi-squared tests were used for categorical variables and a t-test for the mean age to calculate p-values to determine statistically significant differences between demographic variables in the ICU and non-ICU admissions. Data presented as absolute counts, mean (SD) or percentages; ICU: Intensive Care Unit; WIMD: Welsh Index of Multiple Deprivation 2019.

When examining crude 12-month mortality, we observed that in the low HFRS and low CCI categories mortality was significantly higher in the patients admitted to the ICU in all age groups. In patients over 70 years of age, this gap between patients admitted or not admitted to the ICU has closed in the intermediate HFRS and CCI groups and disappeared in the high HFRS and high CCI groups (Supplementary Figure S1 and S2). In patients between 50 and 70 years of age the gap only closed in the high HFRS and CCI groups (Supplementary Figure S1 and S2). We did not observe similar patterns using the eFI score (Supplementary Figure S3).

#### Association between eFI, HFRS and CCI

The cross tabulations for eFI, HFRS and CCI are included in the supplementary material (Supplementary Table S2–S4). Chi-squared tests for independence indicated that the eFI, HFRS and CCI were all associated (p < 0.001). Cramer’s V showed the strength of association was similar amongst all the tools, but strongest between the eFI and HFRS (0.255) and weaker between the CCI and frailty tools (eFI: 0.206, HFRS: 0.217) (Supplementary Table S5).

### Risk factors of mortality at different time-points

Multivariate logistic regression analysis based on the two frailty indices and the comorbidity index consistently had an increased risk of inpatient, 6-month and 1-year mortality for individuals with an ICU admission. Similarly, in all models, advancing age, increased frailty and comorbidity burden together with residence in the most affluent areas affected short- and long-term mortality. The estimated AUC revealed that each model had only moderate discriminatory power for predicting mortality. For predicting inpatient deaths, the CCI and HFRS based models were similar, however for longer term outcomes the CCI based model was superior to the frailty scores (Tables 2, 3, 4).

**Table 2:** Odds Ratios (ORs) for the adjusted multilevel multivariate logistic regression inpatient mortality analyses.

	CCI	eFI	HFRS
ICU admission	2.624 (2.505, 2.749)	2.669 (2.55, 2.794)	2.301 (2.197, 2.411)
**Age category (years, baseline 18–49)**			
50–59	1.982 (1.782, 2.205)	2.278 (2.049, 2.532)	2.070 (1.861, 2.302)
60–69	2.999 (2.731, 3.294)	3.670 (3.341, 4.03)	3.208 (2.921, 3.523)
70–79	4.398 (4.021, 4.811)	5.598 (5.113, 6.13)	4.427 (4.048, 4.842)
80 +	8.665 (7.938, 9.458)	10.997 (10.051, 12.033)	7.422 (6.794, 8.108)
**Sex (baseline—Female)**			
Male	1.034 (1.004, 1.066)	1.105 (1.073, 1.138)	1.119 (1.087, 1.153)
**Charlson index (baseline—less than 1)**			
(1–10)	1.154 (1.107, 1.204)	–	–
> 10	2.501 (2.409, 2.596)	–	–
**electronic Frailty Index (baseline–fit)**			
Mild	–	1.085 (1.040, 1.133)	–
Moderate	–	1.128 (1.078, 1.181)	–
Severe	–	1.165 (1.103, 1.231)	–
**HFRS (baseline – Low risk)**			
Intermediate	–	–	2.648 (2.548, 2.752)
High	–	–	3.306 (3.165, 3.452)
**WIMD (Baseline—1. Most Deprived)**			
2	0.983 (0.941, 1.028)	0.975 (0.933, 1.018)	0.979 (0.937, 1.024)
3	0.927 (0.885, 0.971)	0.912 (0.872, 0.955)	0.940 (0.898, 0.985)
4	0.977 (0.933, 1.024)	0.962 (0.919, 1.007)	0.981 (0.936, 1.028)
5. Least Deprived	0.959 (0.916, 1.005)	0.953 (0.911, 0.998)	0.955 (0.912, 1.001)
Intercept	0.037 (0.032, 0.043)	0.042 (0.037, 0.049)	0.029 (0.025, 0.035)
**Random effects**			
Variance	0.029 (0.002, 0.056)	0.028 (0.002, 0.055)	0.048 (0.003, 0.093)
–	–	–	–
Observations	107,188	107,188	107,188
Groups (Admission Year)	9	9	9
AUC	0.711 (0.708, 0.715)	0.677 (0.674, 0.681)	0.711 (0.708, 0.714)

*ICU* Intensive Care Unit; *CCI* Charlson Comorbidity Index; *eFI* electronic Frailty Index; *HFRS* Hospital Frailty Risk Score; *WIMD* Welsh Index of Multiple Deprivation 2019; *AUC* Area Under the Curve.

**Table 3:** Odds Ratios (ORs) for the adjusted multilevel multivariate logistic regression 6-month mortality analyses.

	CCI	eFI	HFRS
ICU admission	1.670 (1.597, 1.746)	1.730 (1.657, 1.807)	1.450 (1.388, 1.516)
**Age category (years, baseline 18–49)**			
50–59	2.316 (2.126, 2.523)	2.725 (2.505, 2.965)	2.522 (2.316, 2.746)
60–69	3.442 (3.189, 3.715)	4.346 (4.028, 4.688)	3.897 (3.613, 4.204)
70–79	4.871 (4.527, 5.241)	6.369 (5.915, 6.858)	5.231 (4.865, 5.625)
80 +	9.561 (8.902, 10.269)	12.174 (11.31, 13.104)	8.629 (8.032, 9.271)
**Sex (baseline—Female)**			
Male	1.113 (1.084, 1.144)	1.205 (1.174, 1.237)	1.217 (1.185, 1.250)
**Charlson index (baseline—less than 1)**			
(1–10)	1.208 (1.165, 1.252)	–	–
> 10	3.178 (3.074, 3.285)	–	–
**electronic Frailty Index (baseline—fit)**			
Mild	–	1.111 (1.071, 1.153)	–
Moderate	–	1.194 (1.147, 1.243)	–
Severe	–	1.284 (1.222, 1.350)	–
**HFRS (baseline—Low risk)**			
Intermediate	–	–	2.485 (2.407, 2.566)
High	–	–	3.125 (3.009, 3.245)
**WIMD (Baseline—1. Most Deprived)**			
2	1.007 (0.968, 1.049)	0.996 (0.958, 1.036)	1.001 (0.962, 1.042)
3	0.938 (0.900, 0.978)	0.919 (0.883, 0.957)	0.948 (0.910, 0.988)
4	1.008 (0.966, 1.051)	0.986 (0.946, 1.027)	1.007 (0.965, 1.050)
5. Least Deprived	0.939 (0.900, 0.980)	0.933 (0.896, 0.972)	0.934 (0.895, 0.974)
Intercept	0.064 (0.057, 0.071)	0.074 (0.067, 0.083)	0.055 (0.048, 0.063)
**Random effects**			
Variance	0.018 (0.001, 0.035)	0.017 (0.001, 0.033)	0.031 (0.002, 0.061)
–	–	–	–
Observations	107,188	107,188	107,188
Groups (Admission Year)	9	9	9
AUC	0.729 (0.727, 0.733)	0.683 (0.680, 0.687)	0.714 (0.712, 0.718)

*ICU* Intensive Care Unit; *CCI* Charlson Comorbidity Index; *eFI* electronic Frailty Index; *HFRS* Hospital Frailty Risk Score; *WIMD* Welsh Index of Multiple Deprivation 2019; *AUC* Area Under the Curve.

**Table 4:** Odds Ratios (ORs) for the adjusted multilevel multivariate logistic regression 1-year mortality analyses.

	CCI	eFI	HFRS
ICU admission	1.537 (1.471, 1.607)	1.613 (1.545, 1.684)	1.328 (1.271, 1.387)
**Age category (years, baseline 18–49)**			
50–59	2.374 (2.191, 2.571)	2.782 (2.572, 3.01)	2.612 (2.413, 2.828)
60–69	3.472 (3.232, 3.729)	4.340 (4.044, 4.659)	3.985 (3.713, 4.277)
70–79	5.094 (4.757, 5.455)	6.490 (6.057, 6.954)	5.514 (5.152, 5.901)
80 +	10.613 (9.927, 11.346)	12.869 (12.013, 13.786)	9.454 (8.84, 10.111)
**Sex (baseline—Female)**			
Male	1.152 (1.122, 1.184)	1.256 (1.224, 1.29)	1.264 (1.231, 1.298)
**Charlson index (baseline—less than 1)**			
(1–10)	1.267 (1.224, 1.312)	–	–
> 10	3.466 (3.354, 3.583)	–	–
**Electronic Frailty Index (baseline – fit)**			
Mild	–	1.152 (1.111, 1.195)	–
Moderate	–	1.292 (1.242, 1.344)	–
Severe	–	1.464 (1.393, 1.539)	–
**HFRS (baseline—Low risk)**			
Intermediate	–	–	2.500 (2.424, 2.579)
High	–	–	3.413 (3.287, 3.544)
**WIMD (Baseline—1. Most Deprived)**			
2	0.988 (0.949, 1.027)	0.978 (0.941, 1.016)	0.981 (0.943, 1.02)
3	0.924 (0.887, 0.963)	0.906 (0.87, 0.943)	0.934 (0.897, 0.974)
4	0.979 (0.939, 1.021)	0.958 (0.919, 0.998)	0.978 (0.938, 1.02)
5. Least Deprived	0.933 (0.895, 0.973)	0.929 (0.892, 0.967)	0.925 (0.887, 0.964)
Intercept	0.076 (0.068, 0.085)	0.089 (0.081, 0.099)	0.067 (0.059, 0.077)
**Random effects**			
Variance	0.016 (0.001, 0.03)	0.015 (0.001, 0.029)	0.029 (0.002, 0.057)
–	–	–	–
Observations	107,188	107,188	107,188
Groups (Admission Year)	9	9	9
AUC	0.742 (0.739, 0.745)	0.697 (0.694, 0.701)	0.728 (0.726, 0.732)

*ICU* Intensive Care Unit; *CCI* Charlson Comorbidity Index; *eFI* electronic Frailty Index; *HFRS* Hospital Frailty Risk Score; *WIMD* Welsh Index of Multiple Deprivation 2019; *AUC* Area Under the Curve.

## Discussion

In this population-scale, data linkage study, we examined in-patient and long-term mortality among patients hospitalised with pneumonia (including those admitted to ICU), to investigate the predictive validity of frailty assessment tools derived from administrative data. Our data indicates: that an in-patient hospital admission with pneumonia carries a high risk of death, with two out of five patients dying at 1-year; that eFI, HFRS and CCI identify different cohorts of patients within the ICU and non-ICU groups and has only moderate agreement between each other and that each investigated model has moderate predictive power for short- and long-term mortality, with the CCI based model showing best predictive ability at 1-year.

To the best of our knowledge, this is the largest dataset evaluating long-term outcomes for in-patient hospitalisations with pneumonia in relation to frailty. We examined mortality up until 1-year following hospitalisation. Unsurprisingly, patients admitted to ICU had significantly worse outcomes in the short-term. The biggest difference was observed for in-patient mortality, likely reflecting the impact of acute illness severity and need for invasive organ support. This is consistent with previous reports for pneumonia and sepsis^23,24^. The overall in-patient mortality rates observed in our cohort (23.9%) are higher than a recent report from the US (12%), but similar to mortality rates in a German study from 2010- 11 (22%)^25,26^. Direct comparisons are made challenging by differences in study design, discharge destination and care administrative processes.

Given such an apparent susceptibility to poorer long-term outcomes, patients hospitalised with pneumonia are a relevant cohort in which to investigate characterisation and predictive validity of frailty scores. Since frailty measures which derive from administrative data may avoid the implementation burden of bedside assessment, reliance upon proxy information, and potential for recall bias, we utilised two measures we have previously validated in other NHS cohorts, the electronic frailty index (eFI) and Hospital Frailty Risk Score (HFRS)^10,12,13,27,28^.

Interestingly, we found that the eFI, which is probably closer to the theoretical description of an accumulated deficit frailty model, had only moderate agreement with HFRS categorisation in ICU and non-ICU groups. Most ICU patients were considered at intermediate risk according to HFRS but fit or only mildly frail according to eFI, whereas most non-ICU patients were low risk according to HFRS but had a higher proportion who would be considered moderate or severely frail according to eFI. Comorbidity was distributed evenly between ICU and non-ICU patients.

Differences in characterisation within ICU and non-ICU groups according to frailty and comorbidity are not unexpected. A fair to moderate level of agreement between frailty assessment methods is not uncommon and our very recent direct comparison of hospitalised patients 65 years and over, also identified low correlation between eFI and HFRS^13,28^. Consistent with previous studies, we observed significantly poorer outcomes with higher categories of frailty for eFI and HFRS among hospitalised patients – including those admitted to ICU—with the HFRS discriminating to a slightly greater degree than eFI^5,29^.

Conceptualisation and measurement of frailty using retrospective healthcare records presents challenges for HFRS and eFI, each method is dependent upon the quality of coding; for eFI, the coding of deficits (from clinical features, specific illnesses, disabilities, and laboratory values) in primary care, and for HFRS the identification of specific conditions according to ICD-10 during hospitalisation. Maintaining consistency with a cumulative deficit model (with an absolute predicted risk), the eFI assigns equal weight to each variable, whereas HFRS produces a weighted score^12^. The HFRS may miss elements which are important to consideration of frailty, such as polypharmacy or care requirements^13^. The look back periods studied also differ by design: for eFI this was 10-years, and for HFRS 2-years^28^. This may have led to the exclusion of important conditions in HFRS pre-dating hospital coding; alternatively, HFRS, with a shorter look back period, may have served as a more dynamic measure of immediately relevant conditions. Furthermore, important to note that although both the CCI and HFRS use ICD-10 codes, they express different risks prevalent in the same population. Whilst CCI is derived using ICD-10 codes of 17 comorbidities, the HFRS was developed giving weighting to 109 frailty-related ICD-10 codes depending on their association with frailty^13,21^. In a recent study in heart failure patients only moderate agreement was found between CCI and HFRS, although both scores were predictive of outcome^29^.

A novel aspect of our study is the application of frailty assessment tools derived from administrative data in relation to ICU and non-ICU outcomes in pneumonia. Most previous studies reported intensive care outcomes according to the CFS and only two utilised a frailty index as predictor of long-term outcome: a 43-item tool derived from comprehensive geriatric assessment, and a novel 52-item instrument based upon data from patients admitted to a specialist geriatric ICU^30–32^. Recent study of adult patients aged 18 years and over with pneumonia admitted to Australia / New Zealand intensive care units found that only severe or very severe frailty scores (according to CFS) predicted in-hospital mortality; however, such patients will have been selected and ICU admission agreed on the basis of perceived likelihood of benefit^33^. We found that short and long-term mortality was similar in the patients aged 70 years and over who had high frailty scores, whether they were admitted or not to the ICU. Our data further highlights the challenges faced by critical care teams in their management of an ageing and frail population.

An important further consideration is that eFI and HFRS were developed and validated in patient populations aged 65 years and over. However, the potential to assess physiological reserve and vulnerability to poorer outcomes for all hospitalised adults (and all age cohorts) is hugely appealing, and our study presents novel data with regards to the application of these frailty scores in younger patients. In our cohort, approximately one third of the patients were aged less than 65 and it is in this younger group that the frailty scores and the comorbidity evaluation showed the greatest divergence. Clearly, further work is required to establish how it may be possible to incorporate assessment of comorbidity and “frailty” determined on the basis of primary and/ or secondary care administrative data, among all age cohorts who may be admitted to critical care – and this seems likely to require the development of a new predictive model.

## Limitations

Our study has limitations. The administrative in-patient data lacks detailed information about disease severity and medical treatments; comorbidities are dependent upon reliable ICD-10 coding, and variation in documentation and coding of diagnoses could contribute to measurement error^34^. However, in Wales, routinely collected population-scale EHR data are sufficiently robust and accurate to be used in research or for health services planning^35^. We could not analyse the direct impact of possible differences in treatment intensity during ICU admission, as comparative data are not available currently in the SAIL Databank in the hospital cohort. We were unable to examine long-term outcomes other than mortality, and it would be useful to explore how these frailty indices predict subsequent healthcare utilisation, physical function, disability and quality of life^31,36–38^. The observed higher mortality in the low frailty and comorbidity cohorts indicates that acute physiological disturbance leading to ICU admission will have significant effect on short-term outcomes. On the other hand, recent data, including ours, indicates that following ICU and hospital discharge, acute illness characteristics have diminishing impact on long-term outcomes^36,39–41^. Future research should focus on exploring the utility of combining frailty instruments with age, comorbidity and illness severity data, and explore patient-centred outcomes other than mortality.

## Conclusion

Patients admitted to hospital with pneumonia face a significant mortality risk in all age groups with less than three out of five alive at 1-year. When evaluating frailty assessment tools, HFRS and eFI performed differently in the ICU and non-ICU population, HFRS indicating higher risk amongst the ICU patients. Older patients with high frailty scores appear to have similarly poor outcomes with or without ICU admissions. Our results illustrate that there is value in the identification of frailty using indices derived from administrative data, but further work is needed to investigate how best to integrate such data, and to incorporate further with assessment of severity of acute illness and chronic comorbidity.

## Supplementary Information

Supplementary Information.

## Data Availability

The data that support the findings of this study are available from the Secure Anonymised Information Linkage Databank, but restrictions apply to the availability of these data, which were used under license for the current study, and so are not publicly available. Data are however available from the authors upon reasonable request and with permission of the Secure Anonymised Information Linkage Databank.
