# Supplementary material for: Frailty assessed by administrative tools and mortality in patients with pneumonia admitted to the hospital and ICU in Wales
Source: Sci Rep. 2021 Jun 28;11:13407. doi: 10.1038/s41598-021-92874-w (PMC8239046; doi:10.1038/s41598-021-92874-w)
Supplement: Supplementary file 1 — Supplementary Information. [file 41598_2021_92874_MOESM1_ESM.docx]

**Frailty assessed by administrative tools and mortality in patients with pneumonia admitted to the hospital and ICU in Wales**

**SUPPLEMENTARY MATERIAL**

**Table S1. Demographic differences between the cohorts with and without general practice (GP) data. Chi-squared tests were computed to test for independence.**

|  |  | GP data available for all individuals | |  |  |  |
| --- | --- | --- | --- | --- | --- | --- |
|  |  | No | Yes | chi-squared | degrees of freedom | p-value |
|  | N | 133,604 | 107,188 |  |  |  |
| Sex | Female | 66,680 | 53,630 | 0.366 | 1 | 0.5451 |
|  | Male | 66,924 | 53,558 |  |  |  |
| Age | 18-49 | 13,967 | 11,298 | 1.4436 | 4 | 0.8366 |
|  | 50-59 | 11,227 | 9,063 |  |  |  |
|  | 60-69 | 20,646 | 16,544 |  |  |  |
|  | 70-79 | 32,601 | 26,250 |  |  |  |
|  | 80+ | 55,163 | 44,033 |  |  |  |
| electronic Frailty Index | Fit | 28,706 | 28,706 | N/A - GP data is required to calculate the eFI | | |
|  | Mild | 38,324 | 38,324 |  |  |  |
|  | Moderate | 28,233 | 28,233 |  |  |  |
|  | Severe | 11,925 | 11,925 |  |  |  |
| Hospital Frailty Risk Score | Low | 51,963 | 41,547 | 0.45888 | 2 | 0.795 |
|  | Intermediate | 53,429 | 42,981 |  |  |  |
|  | High | 28,212 | 22,660 |  |  |  |
| Charlson Index | <1 | 43,499 | 34,841 | 0.080599 | 2 | 0.9605 |
|  | "1-10" | 39,646 | 31,842 |  |  |  |
|  | ˃10 | 50,459 | 40,505 |  |  |  |
| WIMD 2019 | 1. Most deprived | 30,335 | 25,265 | 120.59 | 4 | <0.01 |
|  | 2 | 28,917 | 23,795 |  |  |  |
|  | 3 | 26,268 | 20,265 |  |  |  |
|  | 4 | 25,158 | 18,737 |  |  |  |
|  | 5. Least deprived | 22,926 | 19,126 |  |  |  |
| Mortality | In-patient | 31,884 | 25,639 | 0.096301 | 1 | 0.7563 |
|  | 6-months | 50,604 | 40,622 | 0.011089 | 1 | 0.9161 |
|  | 1-year | 58,287 | 46,843 | 0.13309 | 1 | 0.7153 |

**Table S2. Cross tabulation for the electronic Frailty Index (eFI) and the Charlson Comorbidity Index (CCI).**

|  |  |  | eFI |  |  |  |
| --- | --- | --- | --- | --- | --- | --- |
|  |  | Fit | Mild | Moderate | Severe | Sum |
| CCI | <1 | 14,924 | 11,613 | 6,356 | 1,948 | 34,841 |
|  | 1-10' | 7,151 | 12,823 | 8,717 | 3,151 | 31,842 |
|  | >10 | 6,631 | 13,888 | 13,160 | 6,826 | 40,505 |
|  | Sum | 28,706 | 38,324 | 28,233 | 11,925 | 107,188 |

**Table S3. Cross tabulation for the Charlson Comorbidity Index (CCI) and the Hospital Frailty Risk Score (HFRS).**

|  |  |  | HFRS |  |  |
| --- | --- | --- | --- | --- | --- |
|  |  | Low | Intermediate | High | Sum |
| CCI | <1 | 18,397 | 11,684 | 4,760 | 34,841 |
|  | 1-10' | 14,566 | 12,505 | 4,771 | 31,842 |
|  | >10 | 8,584 | 18,792 | 13,129 | 40,505 |
|  | Sum | 41,547 | 42,981 | 22,660 | 107,188 |

**Table S4. Cross tabulation for the electronic Frailty Index (eFI) and the Hospital Frailty Risk Score (HFRS).**

|  |  |  | HFRS |  |  |
| --- | --- | --- | --- | --- | --- |
|  |  | Low | Intermediate | High | Sum |
|  | Fit | 17,507 | 8,950 | 2,249 | 28,706 |
| eFI | Mild | 15,267 | 16,232 | 6,825 | 38,324 |
|  | Moderate | 6,948 | 12,821 | 8,464 | 28,233 |
|  | Severe | 1,825 | 4,978 | 5,122 | 11,925 |
|  | Sum | 41,547 | 42,981 | 22,660 | 107,188 |

**Table S5. Chi-squared tests for independence and Cramer’s V for association strength between the Charlson Comorbidity Index (CCI), electronic Frailty Index (eFI) and Hospital Frailty Risk Score (HFRS).**

|  |  | Chi-squared tests | | |  |
| --- | --- | --- | --- | --- | --- |
| Comorbidity indices | | test statistic | df | p-value | Cramer's V |
| CCI | eFI | 9135.7 | 6 | <0.001 | 0.206 |
| CCI | HFRS | 10106 | 4 | <0.001 | 0.217 |
| eFI | HFRS | 13979 | 6 | <0.001 | 0.255 |

Figure S1. Mortality rates for patients in different HFRS categories admitted to hospital with a Pneumonia or Flu diagnosis between 2010 and 2018, stratified by patients who were admitted to an Intensive Care Unit


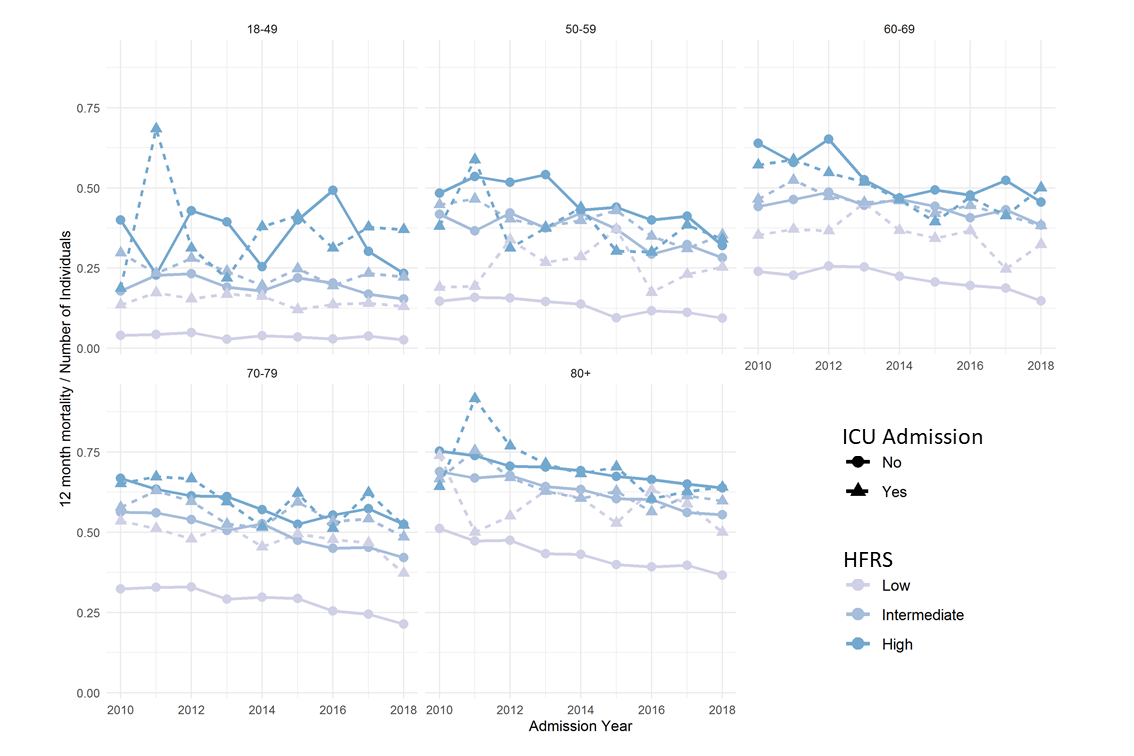


HFRS: Hospital Frailty Risk Score; ICU: Intensive Care Unit

Figure S2. Mortality rates for patients in different CCI categories admitted to hospital with a Pneumonia or Flu diagnosis between 2010 and 2018, stratified by patients who were admitted to an Intensive Care Unit


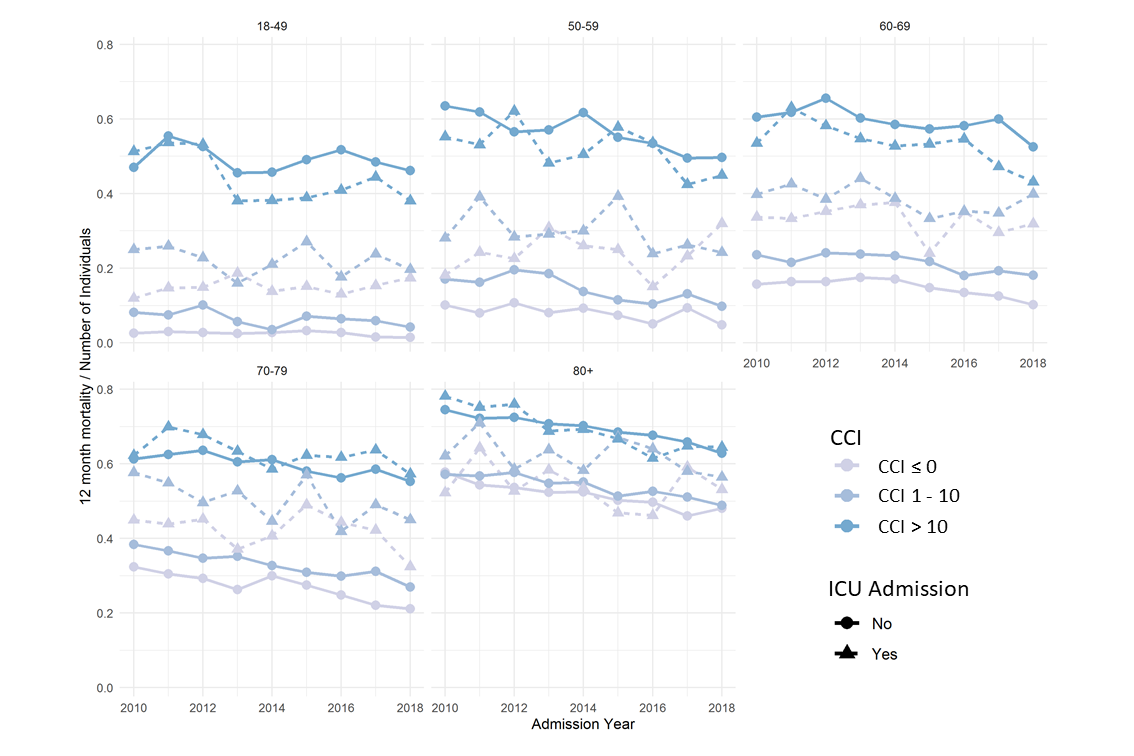


CCI: Charlson Comorbidity Index; ICU: Intensive Care Unit

Figure S3. Mortality rates for patients in different eFI categories admitted to hospital with a Pneumonia or Flu diagnosis between 2010 and 2018, stratified by patients who were admitted to an Intensive Care Unit


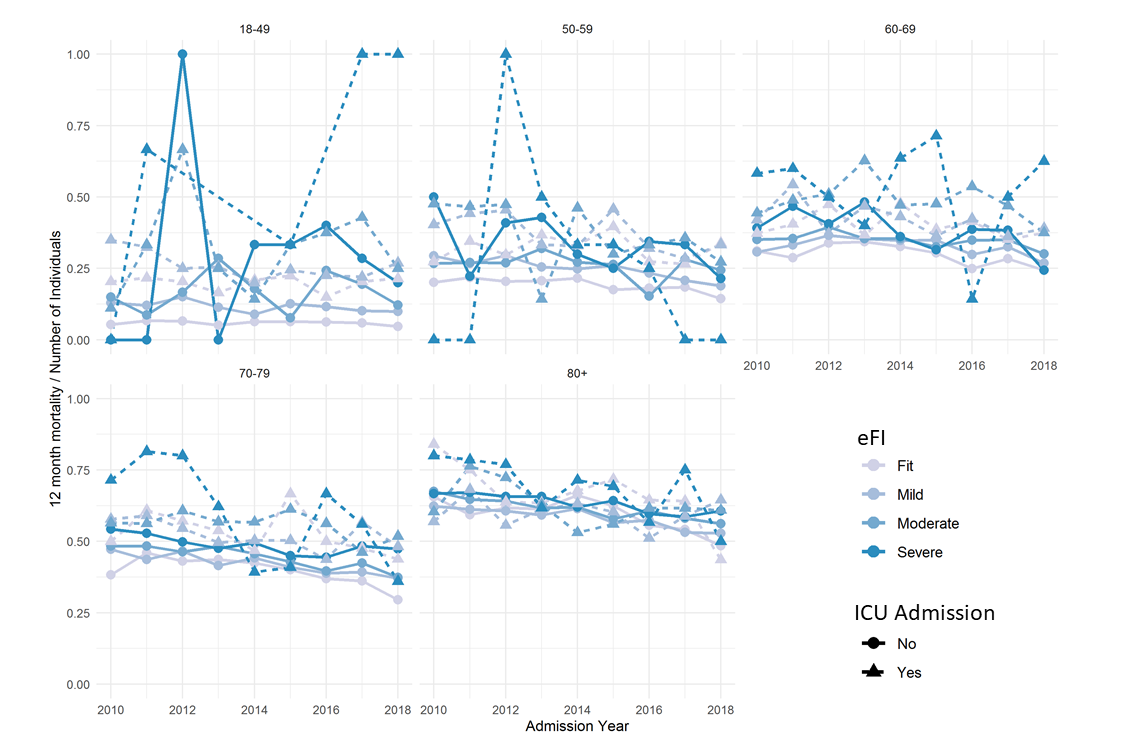


eFI: electronic Frailty Index; ICU: Intensive Care Unit
